# The evidence gap on gendered impacts of performance-based financing among family physicians for chronic disease care: a systematic review reanalysis in contexts of single-payer universal coverage

**DOI:** 10.1186/s12960-020-00512-9

**Published:** 2020-09-22

**Authors:** Neeru Gupta, Holly M. Ayles

**Affiliations:** 1 Department of Sociology, University of New Brunswick, PO Box 4400, 9 Macaulay Lane, Fredericton, New Brunswick E3B 5A3 Canada; 2 Faculty of Management, University of New Brunswick, PO Box 4400, 7 Macaulay Lane, Fredericton, New Brunswick E3B 5A3 Canada

**Keywords:** Physician reimbursement, Gender-based analysis, Health workforce financing, Pay-for-performance, Systematic review

## Abstract

**Background:**

Although pay-for-performance (P4P) among primary care physicians for enhanced chronic disease management is increasingly common, the evidence base is fragmented in terms of socially equitable impacts in achieving the quadruple aim for healthcare improvement: better population health, reduced healthcare costs, and enhanced patient and provider experiences. This study aimed to assess the literature from a systematic review on how P4P for diabetes services impacts on gender equity in patient outcomes and the physician workforce.

**Methods:**

A gender-based analysis was performed of studies retrieved through a systematic search of 10 abstract and citation databases plus grey literature sources for P4P impact assessments in multiple languages over the period January 2000 to April 2018, following the Preferred Reporting Items for Systematic Reviews and Meta-Analyses (PRISMA) guidelines. The study was restricted to single-payer national health systems to minimize the risk of physicians sorting out of health organizations with a strong performance pay component. Two reviewers scored and synthesized the integration of sex and gender in assessing patient- and provider-oriented outcomes as well as the quality of the evidence.

**Findings:**

Of the 2218 identified records, 39 studies covering eight P4P interventions in seven countries were included for analysis. Most (79%) of the studies reported having considered sex/gender in the design, but only 28% presented sex-disaggregated patient data in the results of the P4P assessment models, and none (0%) assessed the interaction of patients’ sex with the policy intervention. Few (15%) of the studies controlled for the provider’s sex, and none (0%) discussed impacts of P4P on the work life of providers from a gender perspective (e.g., pay equity).

**Conclusions:**

There is a dearth of evidence on gender-based outcomes of publicly funded incentivizing physician payment schemes for chronic disease care. As the popularity of P4P to achieve health system goals continues to grow, so does the risk of unintended consequences. There is a critical need for research integrating gender concerns to help inform performance-based health workforce financing policy options in the era of the Sustainable Development Goals.

## Introduction

Governments and healthcare service organizations around the world have increasingly adopted financial incentives to stimulate guideline-based practice for the prevention, diagnostics, and treatment of prevalent diseases. Such incentives, also known as pay-for-performance (P4P), may be offered as added rewards to healthcare practitioners for changes in clinical behaviors in terms of time, services delivered, patients reached, quantity or quality of care, continuity of care, or other established targets to achieve health system goals [1, 2]. The World Health Organization advocates that health system efficiencies could be achieved in countries at all levels of economic development through better incentives for primary care providers and other means of focused financing [3]. Performance incentive schemes have been implemented in several high-income countries and introduced in many low- and middle-income countries, the latter often as donor-supported pilot projects [3, 4]. However, it remains unclear to what extent, if at all, financial incentives positively influence the delivery of care in terms of equitable outcomes by gender or other personal characteristics of either patients or providers [1, 5]. The risk of potential unintended consequences of P4P schemes has tended to be overlooked in the available literature [6].

Enhancing the efficiency and effectiveness of healthcare investments is important in many countries; incentivizing physician payments to improve chronic disease management—versus relying on traditional fee-for-service, capitation, or bundled payments—is an area of increasing attention [2, 7]. Several systematic reviews have examined the impacts of P4P among medical practitioners on different indicators of healthcare processes, costs, and patient outcomes across different contexts and different systems of healthcare financing [1, 2, 4, 5, 7–13]. However, heterogeneity of incentive schemes and evaluation methods has meant there are fragmentation and general deficiency in the evidence base to support the use (or non-use) of incentive reimbursements among physicians to improve primary care for diabetes and other chronic non-communicable diseases (NCDs). Some research has found that physicians may react to incentives differently depending on whether they were for acute or chronic illness [8]. Investing in better management to lessen the impact of chronic NCDs is critical, given that these diseases account for 71% of the total mortality burden worldwide [14]. Moreover, much of the evidence on the impacts of P4P for NCDs pertains to diabetes [6]. Reducing the number of diabetes-related premature deaths is one of the key targets of the international Sustainable Development Goals (SDG) agenda (target 3.4.1). Diabetes and its complications place a substantial long-term burden on health budgets [15]. The greater susceptibility of patients with pre-existing diabetes to COVID-19 has further highlighted the cruciality of addressing diabetes management in health emergencies [14].

While the number of P4P policies continues to increase, along with the number of studies on P4P effects, it is uncertain whether and how P4P is related to better equity in patient outcomes. Some limited research has suggested that certain patient groups, notably older patients and those with multiple chronic NCDs, may benefit less from incentivized care compared to their younger and healthier counterparts [16]. At the same time, rising global prevalence of NCDs and other health challenges run the risk of fueling gender-related health inequalities [14]. Despite the evidence of biological and psychosocial differences between female and male patients in the progression of diabetes and related complications, clinical care guidelines tend not to differentiate by sex or consider gender-sensitive approaches to improve adherence to therapy [17].

Specifically, we are unaware of any reviews evaluating P4P schemes that consider a measure of better gender equity in patient outcomes. Achieving gender equality through strengthened policies and public allocations is another key SDG indicator (target 5.c). Health systems can make important contributions to this SDG by tracking gender inequalities and addressing underlying structural issues, including gender-based assessments of approaches to budgeting [18]. While it is increasingly acknowledged that monitoring sex-specific impacts of health interventions is a critical starting point, sex and gender reporting remains inadequate in health research [19, 20]. Petkovic et al.’s study of recent systematic reviews documented that less than 30% of reviews reported on sex or gender in the results [20]. There is growing recognition that, unless explicit attention is paid in health financing to gender, movement towards meeting population needs can fail to achieve gender balance or improve equity and may even exacerbate gender inequity [21]. This knowledge gap led us to our first research question: Do incentive reimbursements for primary care physicians reflect or even exacerbate gender inequalities in patient-oriented diabetes outcomes, compared to the absence of incentivizing remuneration?

We are further unaware of any P4P schemes adjusted for physicians’ gender or other individual characteristics (aside from practice location), or reviews that consider performance pay in regard to gender wage gaps or other workforce equity measures [5]. Males, including those in medical and other high-paying occupations, have long earned more than their female counterparts. The gender-related pay gaps have not been readily explained by objective labor market characteristics, including educational attainments [22]. Studies from different countries have indicated that female physicians continue to earn on average 13% less than male physicians, after controlling for factors such as specialty and working hours [23, 24]. While health systems are often considered insufficiently responsive to women’s specific health needs, they are also highly dependent on women as providers of care [25]. Women are increasingly predominant in the physician workforce, and specifically the primary care physician workforce, in many countries [24, 26]. As healthcare organizations strive to enhance patient experiences, improve population health, and reduce per capita costs of care, there is also growing recognition that achieving the ultimate goal of a high-performing health system requires improving the work life of service providers—collectively known as the Quadruple Aim for healthcare improvement [27, 28]. The World Health Organization acknowledges that health workforce gender imbalances, including wage differences, are a major challenge for health policymakers to enhance system efficiencies [29]. For one, Hedden et al.’s systematic review presented evidence that female primary care physicians present different clinical practice patterns compared to their male counterparts, including spending more time with each patient and dealing with multiple health issues during a given visit [26]. How differences in physician remuneration mechanisms and financing policies across jurisdictions over time may influence the differences between male and female physicians in observed practice patterns is an important area for a new investigation. This gap incited us to raise our second research question: Do incentive reimbursements reflect or even exacerbate gender inequalities in physician remuneration?

To address these questions, we conducted a reanalysis of a systematic review of the literature on impacts of P4P among primary care physicians for diabetes management and analyzed the evidence concentrating on the extent to which patients’ and/or physicians’ sex/gender is considered or influential in the results to achieve the Quadruple Aim for healthcare improvement. The aim was to enhance the understanding as to whether increasing numbers of women in medicine may drive change in clinical practice patterns without P4P, whether “gender-blind” P4P schemes have a different impact on male versus female patients, and whether such schemes are contributing to gender inequities in professional earnings among providers.

## Methods

### Study design

A reanalysis was conducted using a gender-based analysis approach of the authors’ previously published and unpublished data from a systematic review of P4P evaluation studies for the management of diabetes and other NCDs in publicly funded national health systems [5]. The scope of the review focuses on the contexts of single-payer universal health coverage, thus minimizing the risk of unintended consequences of P4P from physicians gaming the payment system, that is, from physicians potentially moving between health organizations within a jurisdiction to benefit from an incentive, or avoiding high-risk patients altogether to not upset clinical performance metrics [30]. This approach also discounts the specific effects of female medical practitioners potentially sorting out of health organizations with a strong performance pay component or having other characteristics that may be less attractive to women [31]. Substantively, government-funded health systems further have the responsibility in the SDG era to ensure gender-responsive human resources for health (HRH) budgeting, as an important measure to realizing their international commitments to achieving gender equality.

In accordance with other systematic review reanalyses and subanalyses, this study was designed to reconsider a previously published systematic review from a distinct implementation and reporting perspective, thereby allowing for new research questions to be examined in detail while avoiding unwarranted research duplication. The protocol for the present study was published in the PROSPERO prospective register of systematic reviews (registration number CRD42018090021) [32]. Whereas the authors’ original review focused on patient-oriented outcomes before and after the introduction of P4P (e.g., patient morbidity, avoidable hospitalization, premature death) [5], for this study, the primary outcomes of interest are gender equity in P4P effects from the patient and also provider perspectives. The review aligns with the Preferred Reporting Items for Systematic Reviews and Meta-analyses (PRISMA) guidelines [33].

### Data extraction

Studies were eligible to be included in the systematic review if they addressed the question of whether the introduction of physician practice incentives for diabetes management in primary and community care led to improved population health and health system outcomes through some sort of evaluative component. This could include incentives for diabetes-specific care or management of multiple morbidities, from all countries with single-payer health insurance systems.

Ten abstract and citation databases were searched: ABI Inform, Business Source Premier, Canadian Business and Current Affairs, Cochrane Library, EconLit, PAIS, PubMed, Scopus, SocIndex, and Sociological Abstracts. Free text and formal search terms and filters were translated to respect database-specific requirements, with the advice and assistance of library professionals. Several Medical Subject Headings (MeSH) terms and combinations were used to identify the intervention [including “pay#for#performance,” “incentive reimbursement*,” “value#based purchasing,” “performance pay*,” “merit pay*.” and related nomenclature] and the health condition of interest [“diabetes mellitus,” “diabetes,” “hyperglyc*,” “prediabetes,” “dysglyc*,” and related nomenclature]. Reference lists of systematic reviews on the topic that were found during the database searches [1, 2, 4, 7–13] as well as of selected global health literature sources were further hand-searched [3].

Eligible studies included those published in English, French, Portuguese, or Spanish between 1 January 2000 and 30 April 2018. Two reviewers independently screened a sample of eligible abstracts and in turn full-text articles, to identify and secure consensus on studies for review inclusion. The country and its health financing arrangement, characteristics of the incentive scheme, study objective, provider and patient populations, data gathering techniques, comparison groups, and outcomes measured were recorded. The full eligibility criteria and search strategy, which were guided by a Population, Intervention, Comparison, Outcomes, and Study (PICOS) design framework, are available elsewhere in the original review and related protocol [5, 34].

### Data analysis

For this analysis, we developed gender-based analysis grading criteria for the retrieved records. Each study’s contents were vetted distinguishing between “sex” (a biological/physiological characteristic distinguishing males from females) and “gender” (the roles, behaviors, activities, and attributes that a given society may construct or consider appropriate for men and women) [20]. Studies were categorized by five items based on the level of inclusion and reporting of sex and gender data and analysis, pertaining to both patients and providers (Table 1). Simple mentions of the terms sex or gender as statistical control variables were assigned lower scores, while discussions of gender perspectives in the narrative of the results were given higher scores.

**Table 1:** Evaluation grid for consideration of sex and gender in P4P impact assessments

**Item 1:** Relates sex and/or gender in the **study design**	
2 = Methods describe that the analysis will be disaggregated by sex of both patients and providers	
1 = Methods describe that the analysis will be disaggregated by sex of either patients or providers (not both)	
0 = No sex disaggregation described	
**Item 2:** Includes **disaggregated data by sex of the patient** in the results	
2 = Results include sex-disaggregated data for patients in the **P4P assessment**	
1 = Results include **only descriptives** of sex-disaggregated data (e.g., general demographic characteristics of the patient population in tables/figures)	
0 = No sex-disaggregated patient data presented in the results	
**Item 3:** Considers **gender perspective of the patient** as part of the P4P assessment	
2 = Narrative substantively discusses the influence of P4P in gender-based analysis from an **equity perspective** (in the results, discussion, and/or conclusion)	
1 = Narrative only describes how sex and other identity factors impacted on patient outcomes in the results (i.e., minimal attention to gender as relevant to P4P)	
0 = No mention of patient sex/gender in the results, discussion, or conclusion	
**Item 4:** Includes **disaggregated data by sex of the provider** in the results	
2 = Results include sex-disaggregated data for providers in the **P4P assessment**	
1 = Results include **only descriptives** of sex-disaggregated data (e.g., general demographic characteristics of the provider workforce in tables/figures)	
0 = No sex-disaggregated provider data presented in the results	
**Item 5:** Considers **gender perspective of the provider** as part of the P4P assessment	
2 = Narrative substantively discusses the influence of P4P in gender-based analysis from an **equity perspective** (in the results, discussion, and/or conclusion)	
1 = Narrative only describes how sex and other identity factors impacted on provider outcomes in the results (i.e., minimal attention to gender as relevant to P4P)	
0 = No mention of provider sex/gender in the results, discussion, or conclusion	

Two reviewers independently extracted and graded sex and gender reporting information from a sample of eligible full-text articles, with any disagreements resolved by consensus. Articles that received a non-zero score in terms of analyzing P4P from a gender perspective (items 3 and 5) were included in the narrative synthesis of the results.

Building on the authors’ previous work, the quality of the evidence reported in the studies was evaluated following the Grading of Recommendations, Assessment, Development and Evaluations (GRADE) approach for complex social interventions [35], with a letter grade assigned to each study based on two predetermined criteria. The evidence was narratively synthesized in terms of the following:

- *Outcome relevance*: the study measured different dimensions for achieving the Quadruple Aim, notably in terms of improvement of outcomes in relation to patient-oriented care (e.g., fewer complications of chronic disease and other measures that matter to patients), population health (e.g., lower rate of onset of major chronic diseases, fewer premature deaths), healthcare costs (e.g., fewer hospital bed days), and/or work life of providers (e.g., pay equity, fewer burnouts, fewer early retirements) [27, 35].
- *Methodological rigor*: the study utilized population-generalizable data and assessment techniques accounting for potential selection bias and unobservables (e.g., models for analyzing endogenous treatment effects of guideline-based diabetes care) [5].

Because of the heterogeneity of the outcomes and analytical approaches under review, performing a meta-analysis was not possible [5].

## Results

### Article retrieval and inclusion

**Fig. 1:**
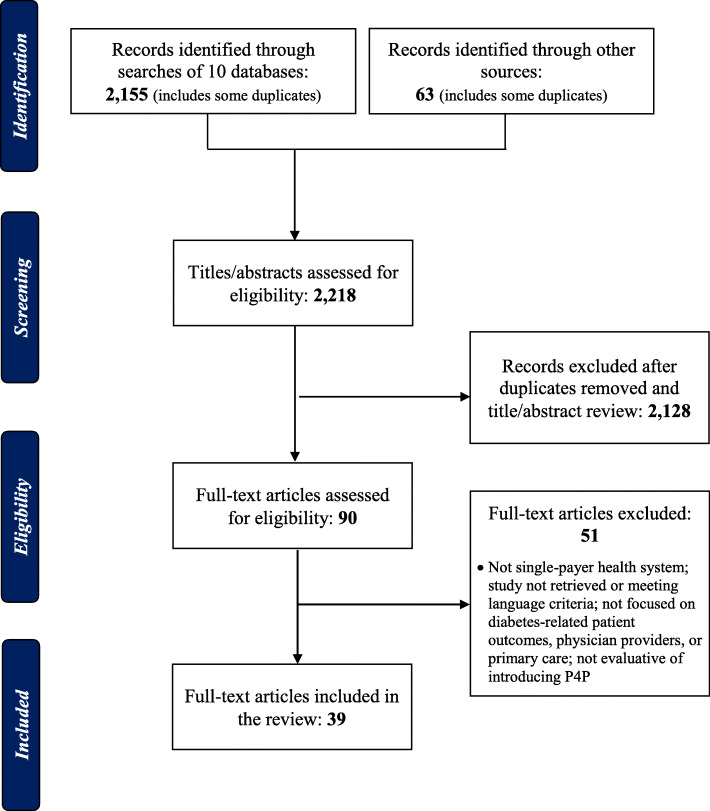
Flow chart for the selection of studies included in the systematic review reanalysis of gendered impacts of P4P for diabetes management in single-payer national health systems, January 2000 to April 2018. Source: Adapted from [5]

A total of 2218 records were initially retrieved: 2155 records from the ten electronic databases plus 63 records from hand searches. In the first step, 2128 duplicates and other records were removed based on the title and abstract screening. Following this screening, 90 articles were retained for full-text review, of which 51 were eventually screened from further consideration. This process left for analysis 39 articles evaluative of introducing P4P among physicians for diabetes and NCD management in primary and community care [5]. A PRISMA depiction of the flow of information is found in Fig. 1.

The studies covered eight unique P4P interventions in seven countries with single-payer health insurance: Australia, Canada (two provincial-level schemes), Denmark, Italy, Sweden, Taiwan, and the United Kingdom [5]. The characteristics of the eight schemes are described in Table 2. Many of the studies used administrative health data sources for the evaluation analyses, typically considered complete and population-representative given the focus on single-payer systems. The full references of the 39 articles reviewed are listed in the Appendix.

**Table 2:** Characteristics of the P4P schemes for diabetes management in single-payer national health systems

Study location	Intervention description	Year introduced
Australia	Bonuses (higher in rural areas) for enrolment and compliance with guidelines for diabetes care, asthma care, and cancer screening	2001
Canada—province of British Columbia	Annual bonus for fee-for-service physicians for compliance with guideline-informed care for two or more targeted chronic conditions	2007
Canada—province of New Brunswick	Annual bonus for fee-for-service family physicians for compliance with diabetes care guideline	2010
Denmark	Annual bonus for compliance with diabetes care guideline	2007
Italy—Emilia-Romagna region	Special payments for guideline-based diabetes care	2003
Sweden—Västra Götaland county	Bonuses for registration of patients with diabetes and achievement of clinical care targets	2011
Taiwan	Bonuses for physician enrolment following diabetes care training plus additional payments for compliance with patient care guideline and performance metrics	2001
United Kingdom	Point-based bonus system among general practices for performance metrics in areas of clinical care, practice organization, and patient experience	2004

Source: Adapted from [5]

### Reporting of sex/gender in P4P assessments

**Fig. 2:**
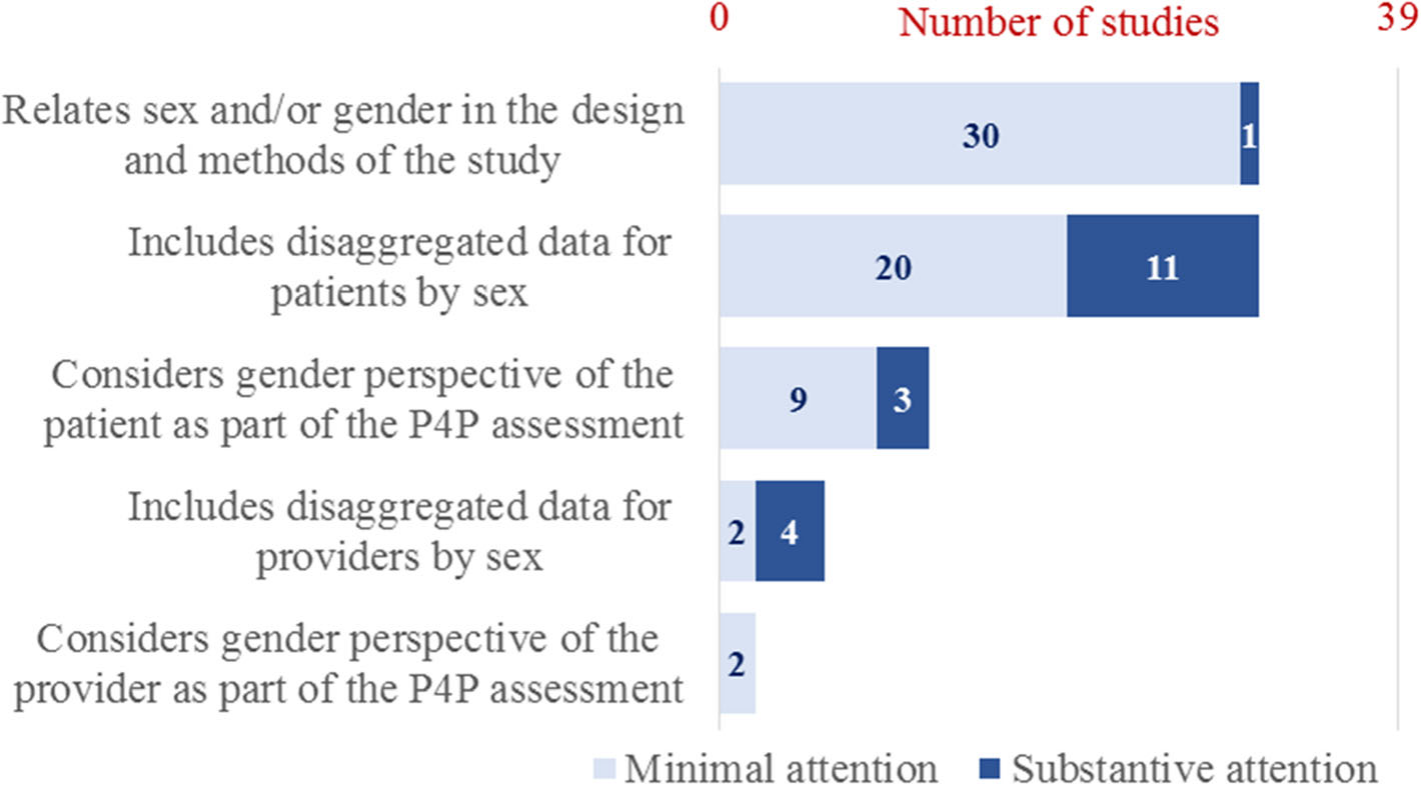
Reporting of sex and gender in P4P impact assessments

Of the 39 studies retained for narrative analysis, 31 (79%) reported that the study considered sex/gender of the patient and/or provider (Fig. 2). Only one substantively detailed that the results would be disaggregated by sex/gender as an integral component of the design. Among the 31 studies indicating any consideration of sex/gender of the patient, two thirds (20 studies or 65%) included only sex-disaggregated descriptives of the patient population among other general demographic characteristics, with the other one third (11 studies or 35%) further reporting sex-disaggregated data in the results of the statistical model assessing the impacts of P4P on patient outcomes. Twelve studies narratively described the sex-disaggregated results, of which nine limited the discussion to the descriptives and three substantively discussed the results in terms of gender-based patient outcomes from an equity perspective.

While most of the studies controlled statistically for the patient’s sex as a demographic variable, few (15%) controlled for the provider’s sex. Of the six studies that did, four presented sex-disaggregated results of the P4P evaluation model. Two discussed the data in terms of sex-specific patterns of provider behaviors. None substantively discussed gender equity from the provider perspective as part of the P4P assessment.

In terms of being able to address our first research question on P4P and gender equity in patient-oriented diabetes outcomes, the 12 studies that narratively discussed sex-disaggregated patient data covered four different P4P schemes: Canada (province of New Brunswick), Italy (Emilia-Romagna region), Taiwan, and the United Kingdom. Eight (67%) of these studies were from Taiwan. For Taiwan, to reduce the risk of bias from multiple reporting of effects of the same intervention, we retained for reporting only the one study classifying gender differences as an integral component of the design as well as the two most recent publications. Table 3 presents the characteristics of the eight studies retained for further analysis following the PICOS framework [6, 36–42].

**Table 3:** Characteristics of the most recent studies narratively discussing the impacts of P4P for diabetes management by patients’ and/or providers’ sex/gender

Author, year	Study location	Population	Comparisons	Outcomes measured	Study analysis method	Sex-disaggregated reporting
LeBlanc et al., 2016 [36]	Canada (New Brunswick)	Adult patients with diabetic glycosylated hemoglobin profile followed by a fee-for-service physician	Patients with/without physician uptake of incentives	− Number of hemoglobin A1c tests − Mean hemoglobin A1c levels	Linear and logistic regression mixed models of linked administrative and laboratory blood test records	− Sex of the patient − Sex of the physician
Lippi Bruni et al., 2009 [37]	Italy (Emilia-Romagna)	Adult patients with type 2 diabetes based on diagnostic profile	Patients with/without physician uptake of incentives, by the presence/absence of a regional P4P scheme	− Hyperglycemic hospital emergency admissions	Multilevel modeling of linked administrative health and hospital records	− Sex of the patient − Sex of the physician
Iezzi et al., 2014 [6]	Italy (Emilia-Romagna)	Adult patients with type 2 diabetes based on drug utilization and specialized care referral profiles	Patients with/without physician uptake of incentives, by the presence/absence of a regional P4P scheme	− Hospitalization for long-term diabetes complications: renal, eye, neurological, and circulatory disorders − Hospitalization for short-term diabetes complications: diabetic ketoacidosis, hyperosmolarity, and coma	Poisson regression models with fixed and random effects specifications of linked longitudinal health administrative records	− Sex of the physician
Yuan et al., 2014 [38]	Taiwan	Adult patients with type 2 diabetes having participated in a clinical evaluation program under P4P	Patients’ length of participation in a diabetes education program	− Diabetes self-management practices − Changes from baseline in hemoglobin A1c levels	Multilevel linear regression modeling of longitudinal program records	− Sex of the patient
Hsiesh et al., 2017 [39]	Taiwan	Patients with type 2 diabetes based on diagnostic profile with comorbid cancer	Patients with/without physician enrolment in P4P	− All-cause mortality − Diabetes-related mortality − Cancer mortality	Multiple regression analysis with propensity score matching of case and control cohorts of linked administrative health records, deaths registry, and cancer registry	− Sex of the patient
Pan et al., 2017 [40]	Taiwan	Patients with newly diagnosed type 2 diabetes based on diagnostic profile	Patients with/without physician enrolment in P4P	− Physician Continuity of Care Index (COCI) − All-cause mortality	Multiple regression analysis with propensity score matching of case and control cohorts of linked administrative health records	− Sex of the patient
Crawley et al., 2009 [41]	United Kingdom (England)	Adults reporting physician-diagnosed diabetes, heart disease, or hypertension	Patients’ occupational group	− Hemoglobin A1c, blood pressure, and cholesterol levels − Use of medications	Multiple regression analysis of annual household survey data including interviews and direct physical measures	− Sex of the patient
Millet et al., 2009 [42]	United Kingdom	Adult patients with type 1 or type 2 diabetes according to medical records	Patients with/without selected comorbid conditions	− Hemoglobin A1c, blood pressure, and cholesterol levels	Multilevel modeling of longitudinal primary care records from a representative sample of general practices	− Sex of the patient

In terms of addressing our second research question on P4P and gender equity in physician remuneration, the two studies that substantively discussed sex-disaggregated provider data in relation to the P4P assessment results were both from Italy. The characteristics of both studies are found in Table 3 [6, 37].

Among the eight studies narratively discussing sex/gender results among patients and/or providers, the number of records on diabetes patients totaled more than 800 000 (ranging from a survey sample of 1173 to a whole-population assessment of 396 838) (Table 4). Most (63%) of the studies did not report the number of providers captured in the data.

**Table 4:** Assessment scores for outcome relevance and methodological quality of the studies included in the review narratively discussing the impacts of P4P by patients’ and/or providers’ sex/gender

	Number of patients with diabetes in the study	Number of providers in the study	Outcome measures	Methods			Assessment
				Selection bias	Study design	Confounders	
LeBlanc et al. [36]	83 580	583	C	B	C	C	Partial evaluation
Lippi Bruni et al. [37]	164 574	2 938	B	A	A	A	Full evaluation
Iezzi et al. [6]	164 574	2 990	A	A	A	A	Full evaluation
Yuan et al. [38]	2 022	n.r.	C	C	C	C	Partial evaluation
Hsieh et al. [39]	2 986	n.r.	A	A	A	A	Full evaluation
Pan et al. [40]	396 838	n.r.	B	A	A	A	Full evaluation
Crawley et al. [41]	1 173	n.r.	C	B	C	C	Partial evaluation
Millet et al. [42]	154 945	n.r.	C	A	C	C	Partial evaluation

*n.r.* not reported. Note: The assessment grid used in the determination of the letter scores for methodological quality is detailed elsewhere [5].

### Impacts of P4P on gender equity in patient outcomes

Based on the quality assessment grid, three of the retained studies discussing sex-disaggregated patient data can be considered full evaluations yielding high-quality evidence on the impacts of P4P on health system outcomes (Table 4). Examples of the narratives describing sex/gender issues in these studies can be found in Table 5. Lippi Bruni et al. reported that patients’ age, insulin dependence, and frequency of visits to diabetes outpatient clinics—but not sex—were the most important determinants of emergency hospitalizations, with the findings robust to different specifications of physician financial incentives in an Italian jurisdiction [37]. In relation to Taiwan’s P4P scheme, Hsiesh et al. reported that all-cause and diabetes-related mortality were lower among patient participants compared to non-participants and that, in terms of confounding factors, female patients with diabetes tended to have a lower risk of cancer mortality than males [39]. Pan et al. reported that patient participants had higher physician continuity than non-participants and that, based on the multiple regression analyses, female patients had significantly higher continuity of care and lower hazard of mortality than male patients [40]. None of the studies discussed sex-specific differences in patient-oriented outcomes by physicians’ P4P uptake.

**Table 5:** Illustrative examples of the reporting of sex/gender in P4P impact studies

Study	Sex-disaggregated reporting
LeBlanc et al. [36]	− Results: “Among patients with baseline A1C levels between 6.5% and 7%, female patients had greater odds than males of receiving at least 2 A1C tests per year. Female physicians for all subgroups of patients were more likely than their male counterparts to order at least 2 A1C tests for their patients” (p. 193). − Discussion: “…our findings suggest that patients followed by female family physicians may have better follow up in diabetes care. This finding is concordant with other studies that found that female physicians prescribe more laboratory tests than males” (p. 195)
Lippi Bruni et al. [37]	− Methods: “Patient demographics include dummies for gender and age classes. Other patient characteristics such as insulin dependence and number of visits to a diabetic outpatient clinic (DOC) are expected to capture severity. We control for GP gender, age and type of practice” (p. 143). − Results: “…the area where the practice is located contributes to the variability between physicians more than the (observed) individual characteristics of the GP himself and of his group of patients. [Regarding the probability of emergency hospitalisations…] as for physician characteristics, age and postgraduate qualifications are not significant, whereas gender is significant and with a positive sign” (p. 145).
Iezzi et al. [6]	− Results: “Individual characteristics of the GP display certain effects [on the risk of diabetes complications], albeit not in a systematic manner. For instance, gender and seniority are not significant and neither practice type nor rural practice location produce any effect” (p. 112).
Yuan et al. [38]	− Background: “The purpose of our study was to investigate how the degree of glycemic control in patients with type 2 diabetes associates with lifestyle interventions as well as sociodemographic factors and further examine the differences by gender. … In addition, we analyzed whether inequalities in health status and disease control existed between genders” (p. 2). − Results: “The average age of the female patients was greater than that of the male patients… Females were less well educated overall in this study population… [and] having physical activities (150 min/weekly) is more associated with the degree of glycemic control in males (*P*=0.003) than in females (푃=0.052)” (p. 3). − Discussion: “The results of this study are intriguing and show that there appear to be sex-based differences in the stage and severity of diabetes... The impact of this health inequality seems to be related to socioeconomic conditions” (p. 8). − Conclusion: “Health inequality is associated with gender and socioeconomic status in Taiwan and is disease-specific” (p. 10).
Hsiesh et al. [39]	− Results: “Regarding other [patient-level] confounding factors, men, older patients, patients with more severe comorbidity and patients with higher baseline density of cancer care tended to have higher risk of all-cause mortality” (p. 5).
Pan et al. [40]	− Methods: “The independent variables consisted of… personal characteristics of the research patients, including gender, age, and monthly salary” (p. e58). − Results: “Compared with female patients, the COCI score of male patients was lower by 0.010 (P<.05)… Male patients showed a higher [hazard ratio] of mortality of 1.75 (95% CI, 1.71-1.80) compared with female patients” (p.e59).
Crawley et al. [41]	− Methods: “logistic regression was performed adjusting for age and gender” (p. 105). − Discussion: “Our findings are consistent with several UK studies have examined equity in quality of care after the introduction of QOF using area-based measures of socioeconomic status… There is increasing evidence that inequities in care between age, gender and ethnic groups have persisted after the introduction of this pay for performance programme in the UK… Policy-makers and purchasers of healthcare should ensure that all such programmes are monitored for possible negative impacts on healthcare equity” (p. 106).
Millet et al. [42]	− Methods: “Patient-level variables were age, sex, ethnicity, neighborhood socioeconomic status (SES), and duration of diabetes” (p. 405). − Results: “Pay for performance was associated with a significantly greater improvement in diastolic blood pressure in men than in women, but this pattern was reversed for A1C” (p. 407). − Conclusions: “Our findings represent a more complete picture of disparities in diabetes management than that derived from national contract data, which lack patient level information on variables such as age, sex, ethnicity, and socioeconomic status and may underestimate variations in care… Our findings suggest that policy makers and health care planners should consider the potential negative impacts of pay for performance incentives on health care disparities” (p. 408).

Among the results of the partial evaluations, Yuan et al. systemically disaggregated patient data by sex in their assessment of an outpatient diabetes quality improvement plan operating within Taiwan’s P4P scheme [38]. The authors found that male patients in the plan tended to have better glycemic control, but that age and socioeconomics were more important drivers of reported patient outcomes. In a Canadian province, LeBlanc et al. indicated no sex-specific difference in the likelihood of patients receiving the guideline-based number of A1c tests between patients followed by physicians who claimed the P4P incentive for diabetes management compared to those followed by physicians who had never claimed the incentive over the period of observation [36]. Reporting on the United Kingdom’s Quality and Outcomes Framework (QOF), Millett et al. indicated that female patients with diabetes were more likely to have multiple comorbid conditions and that diabetes patients with comorbid conditions seemingly benefited more from the introduction of P4P in terms of achievement of established targets for blood glucose and cholesterol than those without comorbidity [42]. Crawley et al. did not report results by patients’ sex in their statistical analysis, which focused on the differences across social class groups but substantively discussed the increasing evidence of inequities in care by socioeconomic status and the limited number of studies using individual-level data in the United Kingdom that consider gender and other characteristics potentially related to persistent inequitable outcomes after P4P introduction [41].

### Impacts of P4P on gender equity in provider outcomes

The two full evaluations that narratively discussed sex-disaggregated HRH data in the P4P assessments were both from Italy (Tables 4 and 5). Lippi Bruni et al. reported that higher shares of practitioners’ income received through P4P was associated with significantly reduced adverse outcomes among their patients, but only under schemes requiring adherence to clinical guidelines [37]. The authors also reported that physicians’ sex, but not their age or postgraduate qualifications, was significantly associated with patients’ risk of emergency hospitalization and notably that patients of female physicians had a significantly lower risk. Iezzi et al. also reported a lower risk of potentially avoidable hospitalization for patients followed by practitioners receiving a higher share of their pay through P4P but that practitioners’ sex and other individual characteristics did not produce systematic effects contributing to the risk [6]. Neither of the studies discussed the impacts of physicians’ P4P uptake on sex-specific differences in professional earnings or other work life indicators.

In their partial evaluation of a low-powered scheme, LeBlanc et al. described that female physicians were more likely than their male counterparts to order the guideline-informed number of A1c tests for their patients, independent of P4P participation [36]. Greene noted that 66% of general practitioners included in the Australian study’s sample were male, similar to the national demographic for all GPs [43].

## Discussion

Pay-for-performance among primary care physicians is increasingly used to enhance guideline-based care practices for diabetes mellitus and other prevalent NCDs. As the number of P4P schemes continues to grow, the potential for unintended consequences may also rise [44], which may possibly include exacerbated gender inequalities in health. This review of P4P impact evaluations in single-payer national health insurance systems revealed that the analysis and reporting of sex and gender in P4P assessments remains inadequate. Of the 39 studies narratively reviewed, most (79%) indicated consideration of the sex/gender of the patient and/or provider in the study design, but only one split all the analyses by patients’ sex as an integral component. One quarter (11 or 28%) of the 39 studies reported sex-disaggregated data in the results of the statistical models assessing influences of P4P on patient outcomes, and three (8%) substantively discussed the results. None (0%) included an interaction term of patients’ sex with the P4P treatment variable, thereby precluding interpretation of gendered impacts of the intervention itself. The already limited discussions concentrated on the presence or absence of sex differentials in the patient-level clinical goals (e.g., glycemic control) rather than in the policy option under investigation.

Consideration of gendered outcomes in the physician workforce was even less extensive. Six (15%) of the 39 studies reported controlling statistically for the providers’ sex. None (0%) included an interaction term of physicians’ sex with the P4P treatment variable or considered an outcome relevant to gender equity in the work life of providers.

In other words, we were unable to answer our original research questions as to whether P4P contributes to gender equity in patient and provider outcomes due to a lack of comprehensive consideration of the issue in the available literature. This finding highlights a critical evidence gap to support physician workforce financing policy decisions that may lead to unintentionally aggravated pre-existing gender inequalities. Some limited research, for example, Boeckxstaens et al.’s review of the United Kingdom’s QOF [45], has suggested that male patients may have benefited more from P4P in terms of quality of care than female patients. A descriptive analysis of physician service billings data from a Canadian province indicated that female family physicians have been under-represented in performance-based payments compared to their male counterparts, potentially exacerbating gender pay gaps [46]. The social, cultural, and psychological reasons why women may respond less to P4P remain largely unknown [31, 46, 47]. Overall, P4P impact assessments focusing on gender and other equity dimensions have been substantially less common compared to those investigating cost-effectiveness [45].

The results of this review were consistent with Petkovic et al.’s examination of systematic reviews extracted from the Campbell and Cochrane Libraries, which revealed inadequate reporting of sex and gender in health research and, specifically, a large gap between the mention of sex/gender in studies’ methods section (51–83%) versus reporting on sex/gender in the results section (less than 30%) [20]. Similarly to Petkovic et al. [20], we did not assess whether the terms “sex” (biological) and “gender” (sociocultural) were used appropriately by the studies’ authors, given the challenge of evolving terminology that is often used interchangeably. In contrast, since we did not restrict any of our database searches using sex/gender search terms, our approach was less likely to have potentially missed instances of sex/gender reporting. It is possible, however, that some studies were missed altogether in our searches given the range in terminology for P4P [5].

The lack of acknowledgment of gender bias in scientific publishing could help explain the knowledge and evidence gaps on gendered impacts of performance-based HRH financing. Gender-blindness in health research and across the sciences is increasingly documented as potentially contributing to reinforce existing gender inequalities, related to a wide range of factors, including bias against research on gender bias [25, 48–50]. For instance, while social science research is often seen as central to enhance understanding of equity in health systems [51], a review of bibliometrics in the social sciences found that articles focusing on gender bias were more often published in journals with a lower impact factor than those considering other dimensions of social discrimination [48]. Some peer-reviewed journals have taken a stance to promote research to help inform actions to address persistent gender inequalities and mitigate gender bias in publication processes [25, 52]; however, avoidance of the identification and reduction of bias remains a seemingly acceptable occurrence. Not all published studies included in this review used gender-inclusive language throughout (e.g., referring to physicians’ characteristics as “the GP himself” [Table 5]). Pervasive (unconscious) gender bias has been quantified in peer review and editorial decision-making outcomes, with men reportedly less likely than women to acknowledge the existence of such a bias [49, 53]. Gender imbalances have also been documented in processes of clinical and public health guidelines development, which may impact the attention given to sex- and gender-specific differences in assessing the value of the evidence [54].

### Strengths and limitations of the study

This study presented a critical interpretation of previously reviewed research from the unique and prospectively planned perspective of gender-based analysis. With the growing number of systematic reviews being published every year, the approach contributed to the literature aiming to optimize the use of identified studies on a given issue where there remained considerable unexplained heterogeneity and unreported information to help support decision making (for example, [55, 56]). The study design was intended to shed light on whether publicly funded primary care physician financing policies for chronic disease care were aligned with international commitments for gender-responsive budgeting for gender equality. The dearth of high-quality evidence suggests that research mechanisms to assess government’s accountability in delivering on gender equality remain insufficient.

The present reanalysis, however, inherited some of the limitations of the original review. Most notably, it was restricted to single-payer national health systems, which meant that relatively few countries were included, none of which were low-income or middle-income countries [5]. This design choice was intended to minimize the risk of measuring physicians’ ability to “game” the payment system rather than true performance; however, such concerns have also been raised in the United Kingdom, as regards P4P potentially reflecting distorted “embellishing” of patient diagnosis codes over the quality of care [57]. Performance pay as a mechanism to improve quality of care first emerged in high-income countries, and much of the research on P4P still tends to be siloed by income setting [58]. Given the proliferation of P4P schemes in low- and middle-income countries, coupled with weaker information systems and the more limited research on P4P effectiveness in many of these contexts [4, 59, 60], rigorous empirical assessments are needed of the relationships (if any) between the allocation of limited resources to performance-based payments and consequences for gender equity from countries at all levels of development.

## Conclusions

This systematic review reanalysis through a sex and gender lens weighed the evidence on how publicly funded performance-based physician remuneration policies may be contributing, positively or negatively, to gender equity in health system outcomes—in this case, in the health outcomes among patients living with diabetes and/or in the work environments among physicians providing diabetes care. Performance-based HRH financing is typically conceptualized as a means to strengthen health systems; however, its implementation and evaluation inadequately consider equity issues [61]. The issue of gender equity has been neglected altogether. Despite the growing recognition of the importance of integrating sex and gender in health research, its practice remains uneven [19]. Gender blindness in health systems and health workforce benchmarking and evidence may miss significant opportunities for gender equity promotion [62]. This review underscored that consideration of gendered impacts in either patient-oriented outcomes or work life of providers is largely overlooked in the P4P literature. Measuring and evaluating the inequitable distribution of power and resources by gender and other social strata, as prerequisites to addressing the problem, remain important on the international health agenda, even if national interests may have waned [63]. Our analysis was consistent with the findings elsewhere revealing a paucity of gendered analyses of health financing arrangements [21]. While it is acknowledged that P4P will exercise different impacts on quality and costs of care depending on the structure of the scheme [44], the evidence base on how such payment models may attenuate or exacerbate gender inequities remains surprisingly weak. Research is needed on HRH financing options to better understand how P4P and other physician payment models may have unintended consequences in terms of gender-specific patient and provider outcomes in the longer term.

## Data Availability

Not applicable. No datasets were generated or analyzed.

## Appendix

List of studies included in the systematic review:

### Australia

1. Greene J. An examination of pay-for-performance in general practice in Australia. *Health Serv Res* 2013; 48(4):1415–32.
2. Scott A, Schurer S, Jensen PH, Sivey P. The effects of an incentive program on quality of care in diabetes management. *Health Econ* 2009;18(9):1091-108.
  **Canada**
3. LeBlanc E, Bélanger M, Thibault V, Babin L, Greene B, Halpine S, Mancuso M. Influence of a pay-for-performance program on glycemic control in patients living with diabetes by family physicians in a Canadian province. *Can J Diabetes*, 2017; 1(2):190–96.
4. Lavergne MR, Law MR, Peterson S, Garrison S, Hurley J, Cheng L, McGrail K. A population based analysis of incentive payments to primary care physicians for the care of patients with complex disease. *CMAJ* 2016; 188(15):e375–e383.
5. Hollander MJ, Kadlec H. Incentive-based primary care: cost and utilization analysis. *Perm J* 2015; 19(4):46-56.
  **Denmark**
6. Rudkjøbinga A Vrangbaek K, Birk HO, Andersen JS, Krasnik A. Evaluation of a policy to strengthen case management and quality of diabetes care in general practice in Denmark. *Health Policy*, 2015; 119(8):1023–30.
  **Italy**
7. Iezzi E, Lippi Bruni M, Ugolini C. The role of GP’s compensation schemes in diabetes care: evidence from panel data. *J Health Econ*, 2014; 34:104–20.
8. Lippi Bruni M, Nobilio L, Ugolini C. Economic incentives in general practice: the impact of pay-for-participation and pay-for-compliance programs on diabetes care. *Health Policy* 2009; 90:140-48.
  **Sweden**
9. Ödesjö H, Anell A, Gudbjörnsdottir S, Thorn J, Björck S. Short-term effects of a pay-for-performance programme for diabetes in a primary care setting: an observational study. *Scand J Prim Health Care*, 2015; 33(4):291–97.
  **Taiwan**
10. Hsieh HM, He JS, Shin SJ, Chiu HC, Lee CT. A diabetes pay-for-performance program and risks of cancer incidence and death in patients with type 2 diabetes in Taiwan. *Prev Chronic Dis*. 2017; 14:E88.
11. Hsieh HM, Chiu HC, Lin YT, Shin SJ. A diabetes pay-for-performance program and the competing causes of death among cancer survivors with type 2 diabetes in Taiwan. *Int J Qual Health Care* 2017; 29(4):512–20.
12. Pan CC, Kung PT, Chiu LT, Liao YP, Tsai WC. Patients with diabetes in pay-for-performance programs have better physician continuity of care and survival. *Am J Manag Care* 2017; 23(2): e57–e66.
13. Chen CC, Cheng SH. Does pay-for-performance benefit patients with multiple chronic conditions? Evidence from a universal coverage health care system. *Health Policy Plan* 2016; 31(1):83-90.
14. Chen YC, Lee CT, Lin BJ, Chang YY, Shi HY. Impact of pay-for-performance on mortality in diabetes patients in Taiwan: a population-based study. *Medicine* 2016; 95(27):e4197.
15. Chi MJ, Chou KR, Pei D, et al. Effects and factors related to adherence to a diabetes pay-for-performance program: analyses of a national health insurance claims database. *J Am Med Dir Assoc* 2016; 17(7):613-19.
16. Hsieh HM, Lin TH, Lee IC, et al. The association between participation in a pay-for-performance program and macrovascular complications in patients with type 2 diabetes in Taiwan: a nationwide population-based cohort study. *Preventive Medicine* 2016; 85:53–59.
17. Hsieh HM, Shin SJ, Tsai SL, Chiu HC. Effectiveness of pay-for-performance incentive designs on diabetes care. *Med Care* 2016; 54(12):1063-69.
18. Huang YC, Lee MC, Chou YJ, Huang N. Disease-specific pay-for-performance programs: do the P4P effects differ between diabetic patients with and without multiple chronic conditions? *Med Care* 2016; 54(11):977-83.
19. Lin TY, Chen CY, Huang YT, et al. The effectiveness of a pay for performance program on diabetes care in Taiwan: a nationwide population-based longitudinal study. *Health Policy* 2016; 120(11):1313-21.
20. Lo HY, Yang SL, Lin HH, Bai KJ, Lee JJ, Lee TI, Chiang CY. Does enhanced diabetes management reduce the risk and improve the outcome of tuberculosis? *Int J Tuberc Lung Dis* 2016; 20(3):376-82.
21. Yen SM, Kung PT, Sheen YJ, Chiu LT, Xu XC, Tsai WC. Factors related to continuing care and interruption of P4P program participation in patients with diabetes. *Am J Manag Care* 2016; 22(1):e18-e30.
22. Hsieh HM, Gu SM, Shin SJ, et al. Cost-effectiveness of a diabetes pay-for-performance program in diabetes patients with multiple chronic conditions. *PLoS ONE* 2015; 10(7):e0133163.
23. Hsieh HM, Tsai SL, Shin SJ, Mau LW, Chiu HC. Cost-effectiveness of diabetes pay-for-performance incentive designs. *Med Care* 2015; 53(2):106-15.
24. Tan EC, Pwu RF, Chen DR, Yang MC. Is a diabetes pay-for-performance program cost-effective under the National Health Insurance in Taiwan? *Qual Life Res* 2014; 23(2):687-96.
25. Yu HC, Tsai WC, Kung PT. Does the pay-for-performance programme reduce the emergency department visits for hypoglycemia in type 2 diabetic patients? *Health Policy Plan* 2014; 29(6):732-41.
26. Yuan SP, Huang CN, Liao HC, et al. Glycemic control outcomes by gender in the pay-for-performance system: a retrospective database analysis in patients with type 2 diabetes mellitus. *Int J Endocrinol* 2014; 2014:575124.
27. Lai CL, Hou YH. The association of clinical guideline adherence and pay-for-performance among patients with diabetes. *J Chin Med Assoc* 2013; 76(2):102-7.
28. Chang RE, Lin SP, Aron DC. A pay-for-performance program in Taiwan improved care for some diabetes patients, but doctors may have excluded sicker ones. *Health Affairs* 2012; 31(1):93-102.
29. Cheng SH, Lee TT, Chen CC. A longitudinal examination of a pay-for-performance program for diabetes care: evidence from a natural experiment. *Med Care* 2012; 50(2):109-16.
30. Lee TT, Cheng SH, Chen CC, Lai MS. A pay-for-performance program for diabetes care in Taiwan: a preliminary assessment. *Am J Manag Care* 2010; 16(1):65-69.
  **United Kingdom**
31. Kontopantelis E, Springate DA, Ashworth M, Webb RT, Buchan IE, Doran T. Investigating the relationship between quality of primary care and premature mortality in England: a spatial whole-population study. *BMJ* 2015; 350:h904.
32. Alshamsan R, Lee JT, Majeed A, et al. Effect of a UK pay-for-performance program on ethnic disparities in diabetes outcomes: interrupted time series analysis. *Ann Fam Med* 2012; 10(3):228-34.
33. Oluwatowoju I, Abu E, Wild SH, Byrne CD. Improvements in glycaemic control and cholesterol concentrations associated with the Quality and Outcomes Framework: a regional 2-year audit of diabetes care in the UK. *Diabet Med* 2010; 27(3):354-59.
34. Campbell SM, Reeves D, Kontopantelis E, et al. Effects of pay for performance on the quality of primary care in England. *N Engl J Med* 2009; 361(4):368-78.
35. Crawley D, Ng A, Mainous AG, et al. Impact of pay for performance on quality of chronic disease management by social class group in England. *J R Soc Med* 2009; 102(3):103-7.
36. Millett C, Bottle A, Ng A, et al. Pay for performance and the quality of diabetes management in individuals with and without co-morbid medical conditions. *J R Soc Med* 2009; 102(9):369-77.
37. Millett C, Netuveli G, Saxena S, Majeed A. Impact of pay for performance on ethnic disparities in intermediate outcomes for diabetes: a longitudinal study. *Diabetes Care* 2009; 32(3):404-9.
38. Vaghela P, Ashworth M, Schofield P, Gulliford MC. Population intermediate outcomes of diabetes under pay-for-performance incentives in England from 2004 to 2008. *Diabetes Care* 2009; 32(3):427-29.
39. Millet C, Gray J, Saxena S, et al. Ethnic disparities in diabetes management and pay-for-performance in the UK: the Wandsworth prospective study. *PLoS Med* 2007; 4(6):e191.

## Abbreviations

COCI: Continuity of Care Index
HRH: Human resources for health
NCD: Non-communicable disease
P4P: Pay-for-performance
PICOS: Population, Intervention, Comparison, Outcomes, Study design
PRISMA: Preferred Reporting Items for Systematic Reviews and Meta-Analyses
QOF: Quality and Outcomes Framework
SDG: Sustainable Development Goals
